# Fgf Signaling is Required for Photoreceptor Maintenance in the Adult Zebrafish Retina

**DOI:** 10.1371/journal.pone.0030365

**Published:** 2012-01-26

**Authors:** Sarah Hochmann, Jan Kaslin, Stefan Hans, Anke Weber, Anja Machate, Michaela Geffarth, Richard H. W. Funk, Michael Brand

**Affiliations:** 1 DFG-Center for Regenerative Therapies Dresden, Cluster of Excellence (CRTD), Biotechnology Center, Technische Universität Dresden, Dresden, Germany; 2 Medical Faculty Carl Gustav Carus, Technische Universität Dresden, Dresden, Germany; National University of Singapore, Singapore

## Abstract

Fibroblast growth factors (Fgf) are secreted signaling molecules that have mitogenic, patterning, neurotrophic and angiogenic properties. Their importance during embryonic development in patterning and morphogenesis of the vertebrate eye is well known, but less is known about the role of Fgfs in the adult vertebrate retina. To address Fgf function in adult retina, we determined the spatial distribution of components of the Fgf signaling pathway in the adult zebrafish retina. We detected differential expression of Fgf receptors, ligands and downstream Fgf targets within specific retinal layers. Furthermore, we blocked Fgf signaling in the retina, by expressing a dominant negative variant of Fgf receptor 1 conditionally in transgenic animals. After blocking Fgf signaling we observe a fast and progressive photoreceptor degeneration and disorganization of retinal tissue, coupled with cell death in the outer nuclear layer. Following the degeneration of photoreceptors, a profound regeneration response is triggered that starts with proliferation in the inner nuclear layer. Ultimately, rod and cone photoreceptors are regenerated completely. Our study reveals the requirement of Fgf signaling to maintain photoreceptors and for proliferation during regeneration in the adult zebrafish retina.

## Introduction

Teleost fish possess a tremendous ability to regenerate injured organs [1], [2], [3], [4], [5], [6], [7], [8]. The remarkable capacity in terms of tissue regeneration and the availability of feasible genetic approaches to manipulate adult zebrafish are key benefits in studying the complex molecular mechanisms involved in regeneration *in vivo*. We use the zebrafish retina to study processes during adult retinal degeneration and regeneration. A variety of lesion paradigms including surgical excision, toxin injection, and exposure to intense light have been examined in the past to damage different retinal cell classes [9], [10], [11], [12], [13], [14], [15], [16]. Unlike mammals, the adult zebrafish is able to restore the complex architecture and function of the neural retina following injury, and two different cell sources are involved in the process of retinal neurogenesis and regeneration. One source is the ciliary marginal zone (CMZ), which is located at the periphery of the retina. The cells in the CMZ continually give rise to all types of neurons in the adult zebrafish and thereby provide lifelong retinal growth and neurogenesis. The second neurogenic source comes from the Mueller glia cells (MGC). The MGC are located in the inner nuclear layer (INL) of the retina and are able to generate photoreceptor progenitors, which are restricted to the rod lineage. After injury, MGC are able to give rise to multipotent progenitor cells, which proliferate and substitute all types of neurons to reconstitute the previous tissue architecture [17], [18], [19]. Investigations in zebrafish fin, heart and brain revealed a crucial role for Fgf signaling during homeostasis and regeneration [20], [21], [22], [23], [24]. Furthermore, inhibiting Fgf signaling from prenatal stages onwards causes degeneration of rod cells in the mouse retina. The loss of rods proceeds very slowly and complete loss is observed only after long-term suppression of Fgf signaling in mice that are several months old [25], [26], [27]. Collectively, these studies suggest that Fgf signaling plays a crucial role in tissue homeostasis. Whether Fgf signaling is important for retinal homeostasis and for recovery after injury is not yet known. The aim of our study is to understand the role of Fgf signaling in homeostasis and after injury in the adult zebrafish retina. To this end, we studied the spatial distribution of Fgf components in the adult retina. Our experiments revealed differential expression of specific Fgf receptors, ligands and target genes in the adult retina. Second, we blocked the Fgf signaling to better understand its importance for retinal homeostasis. We found that impaired Fgf signaling causes severe degeneration of the photoreceptor layer. Third, we show that photoreceptor cell loss induced a fast and profound regeneration response, during which Fgf may promote precursor proliferation. Taken together our results suggest that Fgf signaling is required for maintenance of photoreceptor cells in the adult retina and plays a role in proliferation during photoreceptor regeneration.

## Results

### Fgf pathway expression in the adult neural retina

**Figure 1:**
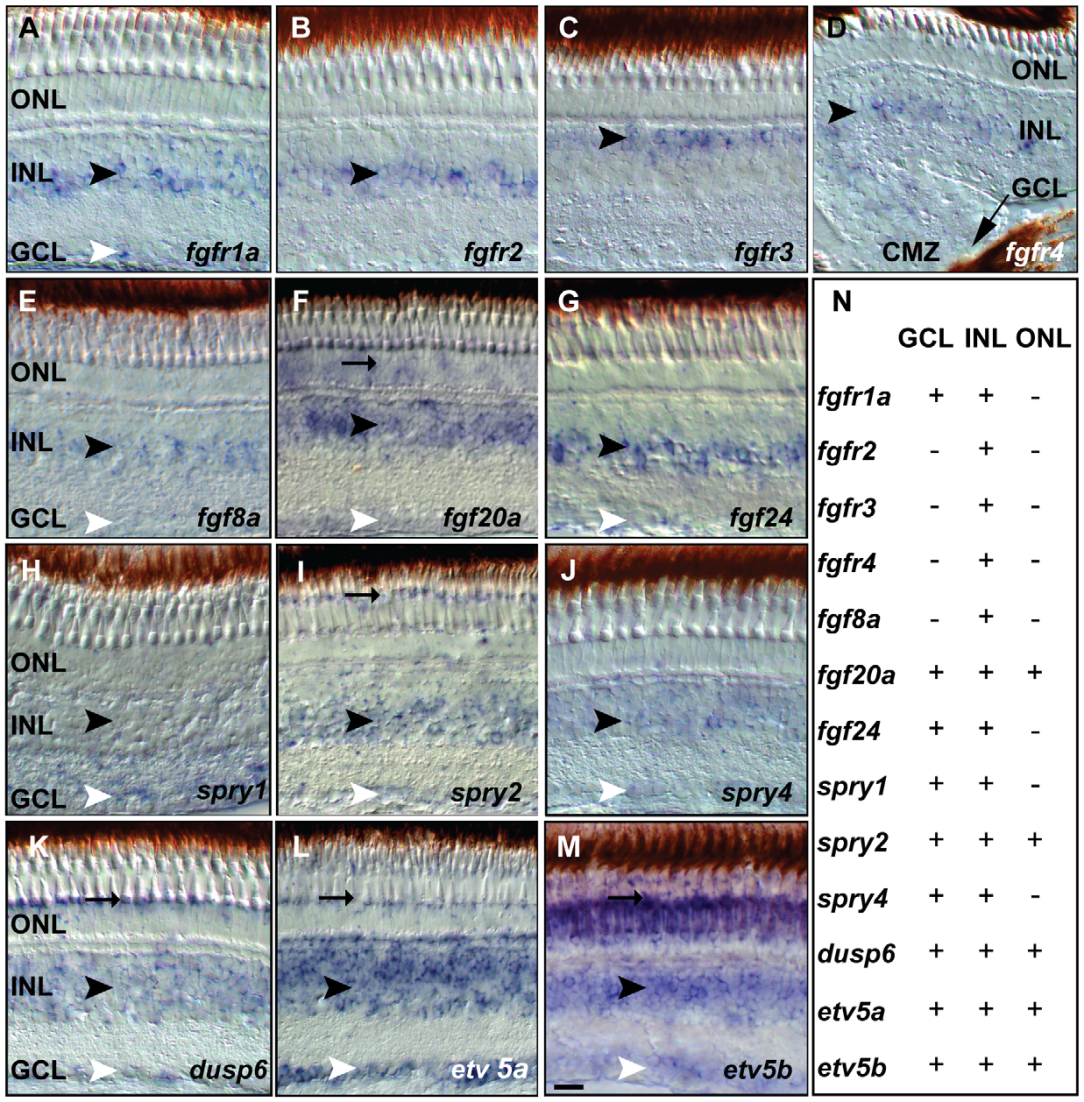
Fgf receptors, ligands and downstream target expression in specific layers of the adult zebrafish retina. **A**) *fgfr1a* expression in the INL and GCL. **B**) *fgfr2* signal in the INL **C**) *fgfr3* expression in the outer part of the INL **D**) *fgfr4* expression in the INL next to the CMZ (black arrow). **E**) *fgf8a* expression in the INL and weakly in the GCL. **F**) *fgf20a* expression in the ONL, INL and GCL. **G**) *fgf24* is detectable in the INL and GCL. **H–M**) Fgf pathway target gene expression. **H**) *spry1* expression in the INL and GCL. **I**) *spry2* signal in POS, INL and GCL. **J**) *spry4* expression in the INL and weakly in the GCL. **K**) *dusp6* expression is strong in the POS, and in the INL and GCL. **L**) Strong *etv5a* expression is found in the POS, INL and GCL. **M**) *etv5b* expression is widespread in the ONL, INL and GCL and enriched in the POS. **N**) Summary of ISH expression data: + expression, − no detectable expression. GCL, ganglion cell layer: white arrowhead; INL, inner nuclear layer: black arrowhead; ONL, outer nuclear layer; POS, photoreceptor layer: black arrow. Scale bar = 20 µm.

The role of Fgf signaling in the adult zebrafish retina is little studied, hence we initially investigated the expression profile of several Fgf receptors (Fgfr), ligands and target genes by *in situ* hybridization (Fig. 1 A–M). Reverse transcriptase (RT-) PCR analysis on cDNA prepared from adult zebrafish eyes revealed the presence of transcripts for *fgfr1a*, *fgfr2*, *fgfr3*, *fgfr4*, *fgf2*, *fgf5*, *fgf7*, *fgf8a*, *fgf8b*, *fgf18*, *fgf18l*, *fgf20a* and *fgf20b* but not of *fgf1*, *fgf6a* and *fgf14* (data not shown) in the adult eye. *In situ* hybridization analysis on cryosections of adult wild-type (WT) zebrafish retina confirmed the expression, which occurs in a layer specific manner. Expression profiles of four of five Fgf receptors are detected in the adult zebrafish retina. *fgfr1a* and *fgfr2* are expressed in the inner half of the INL, whereas *fgfr3* expression is complementary, in the outer half of the INL (Fig. 1A–C). The expression of *fgfr1a*, *fgfr2* and *fgfr3* occurs in central and peripheral parts of the retina, whereas *fgfr4* is mostly expressed in the peripheral ciliary marginal zone (CMZ) and is absent from the central region (Fig. 1D). *Fgfr1b* was not expressed above background levels (not shown). Next, we tested Fgf ligand expression by *in situ* hybridizations and found expression of *fgf8a*, *fgf20a* and *fgf24* in the INL (Fig. 1E–G), of *fgf20a* and *fgf24* in the ganglion cell layer (GCL), and broad *fgf20a* expression in the ONL and in photoreceptor outer segments. We did not detect *fgf2*, *fgf3*, *fgf10a*, *fgf13b*, *fgf17* in the adult retina (data not shown). Several target genes of the Fgf signaling pathway faithfully reflect sites of Fgf signaling in multiple zebrafish tissues, including *spry1*, *spry2*, *spry4*, *dusp6*, *etv5a* and *etv5b* [28], [29]. In the retina, *spry1* and *spry4* are uniformly expressed in the INL and GCL. *spry2*, *dusp6*, *etv5a* and *etv5b* expression is detected in the INL and GCL, and very prominently, in the photoreceptor layer. e*tv5b* is more widely expressed in the photoreceptor layer than the other target genes (Fig. 1H–M); a summary of these results is shown in Fig. 1N.

**Figure 2:**
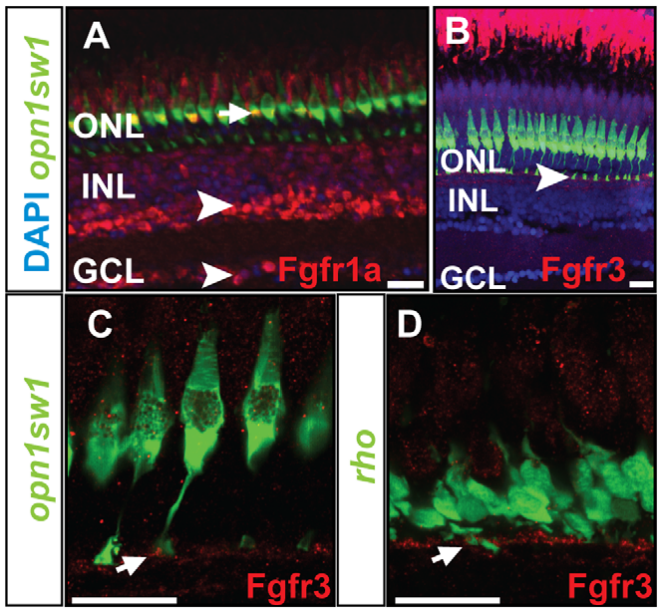
Protein expression pattern of Fgf receptors. **A**) Fgfr1a protein is detected in the photoreceptor layer colocalizing with UV cones (green) (white arrow), INL and GCL (white arrowhead). **B**) Expression of Fgfr3 is detected in the outer part of the INL next to the UV cone synaptic terminals (white arrowhead). **C, D**) Fgfr3 is colocalized with the synaptic terminals of UV cones and rods (white arrows). Scale bars = 20 µm.

We also probed for the presence of the Fgf receptors Fgfr1a and Fgfr3 using immunohistochemistry. In agreement with the *in situ* hybridization analysis, an antibody raised against Fgfr1a detected expression in the INL and GCL, but also in the photoreceptor layer (Fig. 2A). Fgfr3 expression is found in the outer half of the INL, similar to the *in situ* expression pattern (Fig. 2B). Using the transgenic lines *Tg(-5.5opn1sw1:EGFP)kj9* and *Tg(-3.7rho:EGFP)kj2* expressing GFP in ultraviolet – (UV) sensitive cone [30] and rod photoreceptor cells [31], we co-localized Fgfr3 expression to the synaptic terminals of the UV cones and rods, respectively (Fig. 2C–D). Taken together, our results suggest that Fgf signaling pathway components are present and might function in several layers and cell types of the adult zebrafish retina, including its photoreceptors.

### Loss of Fgf signaling leads to rapid death of photoreceptors

**Figure 3:**
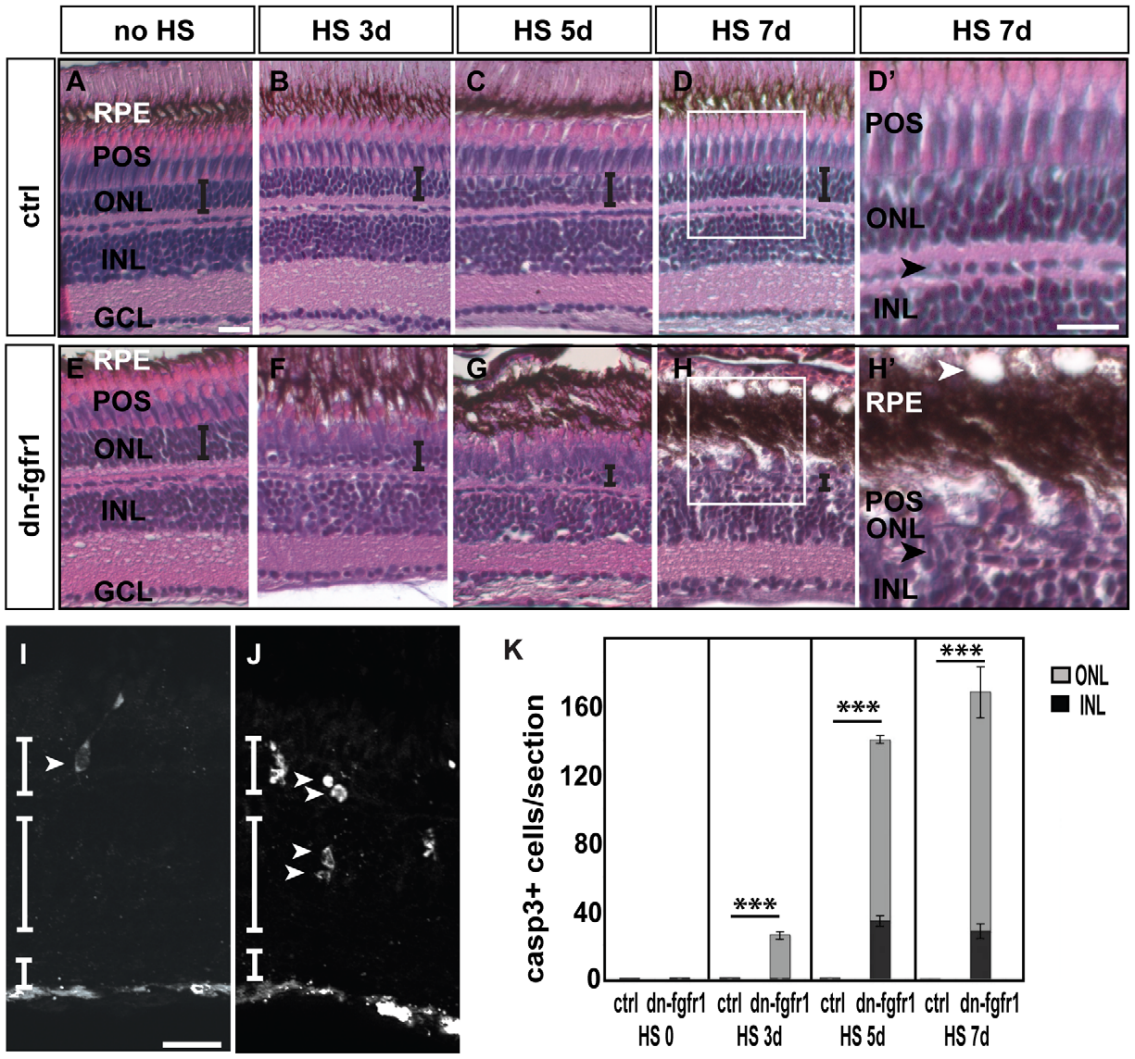
Degeneration of the retina of dn-fgfr1 transgenic animals over time. **A, E**) Control (ctrl) and *Tg(hsp70l:dnfgfr1-EGFP)* (dn-fgfr1) siblings without heat shock (HS) treatment: in both cases no morphological changes were detected **B–D**) In control siblings, the architecture of the retina and the thickness of the ONL (indicated by the error bar) remains unaffected **F–H**) In contrast, in transgenic animals a decrease in organization and in the width of the ONL (indicated by decreasing size of error bars) is observed. **D′, H′**) Insets show the normal structure of control retina and the changes in the retinas of dn-fgfr1 animals. In dn-fgfr1 transgenics, vacuoles appear in the RPE (white arrowhead), and the thickness of the RPE increases. The POS and ONL decrease in thickness. For orientation purposes, the black arrowheads indicate the border of horizontal cells. **I**) An activated Caspase-3 positive cell in the ONL (white arrowhead) after heat shock treatment on three consecutive days. **J**) Activated Caspase-3 positive cells in the INL and ONL (arrowheads) after five days of Fgf signaling inhibition. **G**) Quantifications of activated Caspase-3 positive cells per section over time. In wild-type and transgenic fish without any heat shock, dying cells are not detectable (p = 2,28). After three days of heat shock treatment dying cells are detected in the transgenic fish only in the ONL (grey column) (p = 2,44E-08). After five (p = 1,04E-41) and seven days, an increased number of activated Caspase-3 positive cells are in both the ONL (grey column) and INL (black column) (p = 6,42E-26). Shown are the mean numbers of Casp3+ cells/section. Error bars indicate the standard error of the mean (SEM). p-values: *≤0.05, **≤0.01, ***≤0.001. ctrl, control; HS, heat shock; dn-fgfr1, *Tg(hsp70l:dnfgfr1-EGFP)*. Scale bars = 20 µm.

**Figure 4:**
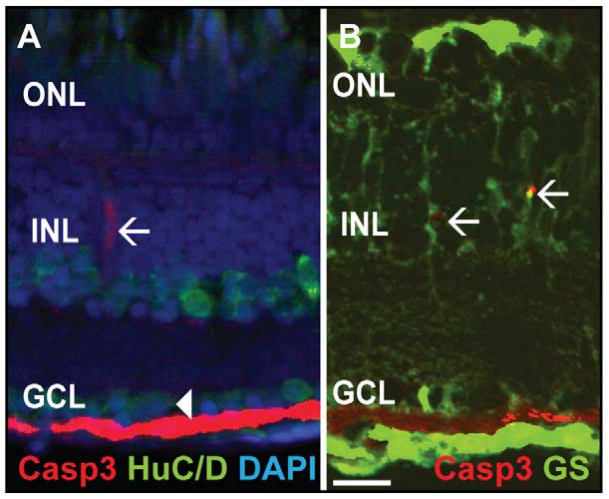
Double labeling of Caspase-3 positive cells. **A**) Casp3 (red) and HuC/D (green), marker for mature neurons such as amacrine and ganglion cells, do not colocalize in the INL (white arrow) and GCL (white arrowhead). **B**) Glutamine synthetase (GS, green), a marker for MGC, colocalizes with Casp3+ (red) cells in the INL (white arrows). Scale bar = 20 µm.

To investigate the functional role of Fgf signaling in the adult zebrafish retina, we focused on its potential role in photoreceptor cells. We used a transgenic line expressing a dominant negative version of receptor 1 (dn-fgfr1) under the control of the zebrafish temperature-inducible *hsp70l* promoter (*Tg(hsp70l:dnfgfr1-EGFP)*). The dn-fgfr1 lacks the intracellular domain, binds to endogenous Fgf receptors and efficiently blocks all Fgf signaling [20]. Adult dn-fgfr1 transgenic fish and wild-type siblings were heatshocked once daily up to seven days. Subsequent histological analysis revealed a rapid and severe disorganization of the retinal structure already within seven days (Fig. 3H). Transgenic fish display a progressive loss of the photoreceptor outer segments (POS) and thinning of the outer nuclear layer (ONL) visibly starting from three days of heat shock treatment (Fig. 3F) and getting more severe over time. After one week of continuously blocked Fgf signaling, photoreceptor cells are almost completely lost (Fig. 3H). This phenotype is more severe in the dorsal and central region of the retina than in the ventral retina (data not shown). In addition, the cellular packing density in the INL decreases, and fusiform-shaped cells appear at the junction to the ONL (Fig. 3H′, black arrowhead), possibly indicating cell migration towards the ONL. In contrast, non-transgenic wild-type control siblings subjected to the same heat shock treatment did not show any sign of degeneration after one week of treatment (Fig. 3A–D). To determine whether Fgf inhibition results in cell death, we used an antibody against activated Caspase-3. After three days of heat shock, a small number of Caspase-3-positive (Casp3+) cells were found in the ONL of *Tg(hsp70l:dnfgfr1-EGFP)* transgenic but not in the ONL of wild-type siblings (Fig. 3I, white arrowhead and data not shown). At seven days, we detected a significant increase in the number of Casp3+ cells in the ONL and a few cells in the INL (Fig. 3J, white arrowhead) of transgenic fish. We did not detect any Casp3+ cells in wild-type siblings. To determine the identity of the Casp3+ cells in the INL of transgenic siblings, we performed antibody stainings for HuC/D, a marker for retinal neurons such as amacrine and ganglion cells. We did not detect any Casp3+/HuC/D+ cells in the INL and GCL (Fig. 4A). Moreover, we performed antibody stainings for glutamine synthetase (GS), a Müller glia cell marker and detect Casp3+/GS+ double labeled MGC in the INL (Fig. 4B), possibly reflecting phagocytosis of cellular debris by MGCs [32]. Quantification of the Casp3+ cells (Fig. 3K) revealed that photoreceptor cells progressively die between three and seven days of disrupted Fgf signaling, predominately in the ONL. The amount of Casp3+ cells in transgenic animals is significantly higher after three or more days of heat shock treatment compared to control fish. Our results showed that rapid photoreceptor degeneration occurs after loss of Fgf signaling.

**Figure 5:**
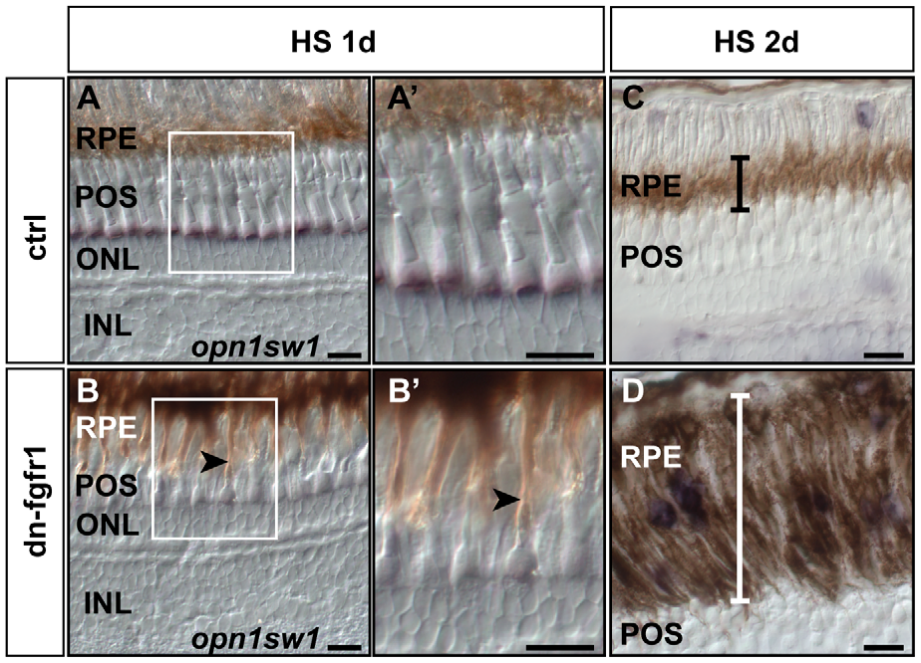
Retinal pigment epithelium is expanded following Fgf signaling inhibition. **A**) After one day of heat shock treatment control siblings do not show any change in the RPE structure. **B**) In transgenic siblings, RPE cells appear more stretched, reaching out to the outer segments of the photoreceptor cells (black arrow). **A′, B′**) At higher magnification, the RPE contacts photoreceptor cells in the POS in the transgenic fish (arrow), while this was not detected in retinas of control fish. **C**) The black error bar indicates the width of the RPE in control siblings. **D**) In contrast, the width of the RPE is highly increased (white error bar) in retinas of dn-fgfr1 fish after two days of heat shock treatment. Scale bars = 20 µm.

Cells of the retinal pigment epithelium (RPE) are also affected early after loss of Fgf signaling. Already after one day of heat shock treatment, RPE cells appear to elongate towards the photoreceptors in transgenic but not in control fish (Fig. 5A–B′), apparently to stay in contact with them. Thus, the RPE layer expanded in size compared to control siblings (Fig. 5C–D) and vacuoles appeared in the RPE (Fig. 3H′). Taken together, we detect a very fast response of the RPE linked to photoreceptor cell death.

### Block of Fgf signaling results in loss of photoreceptor markers and Fgf target gene expression

**Figure 6:**
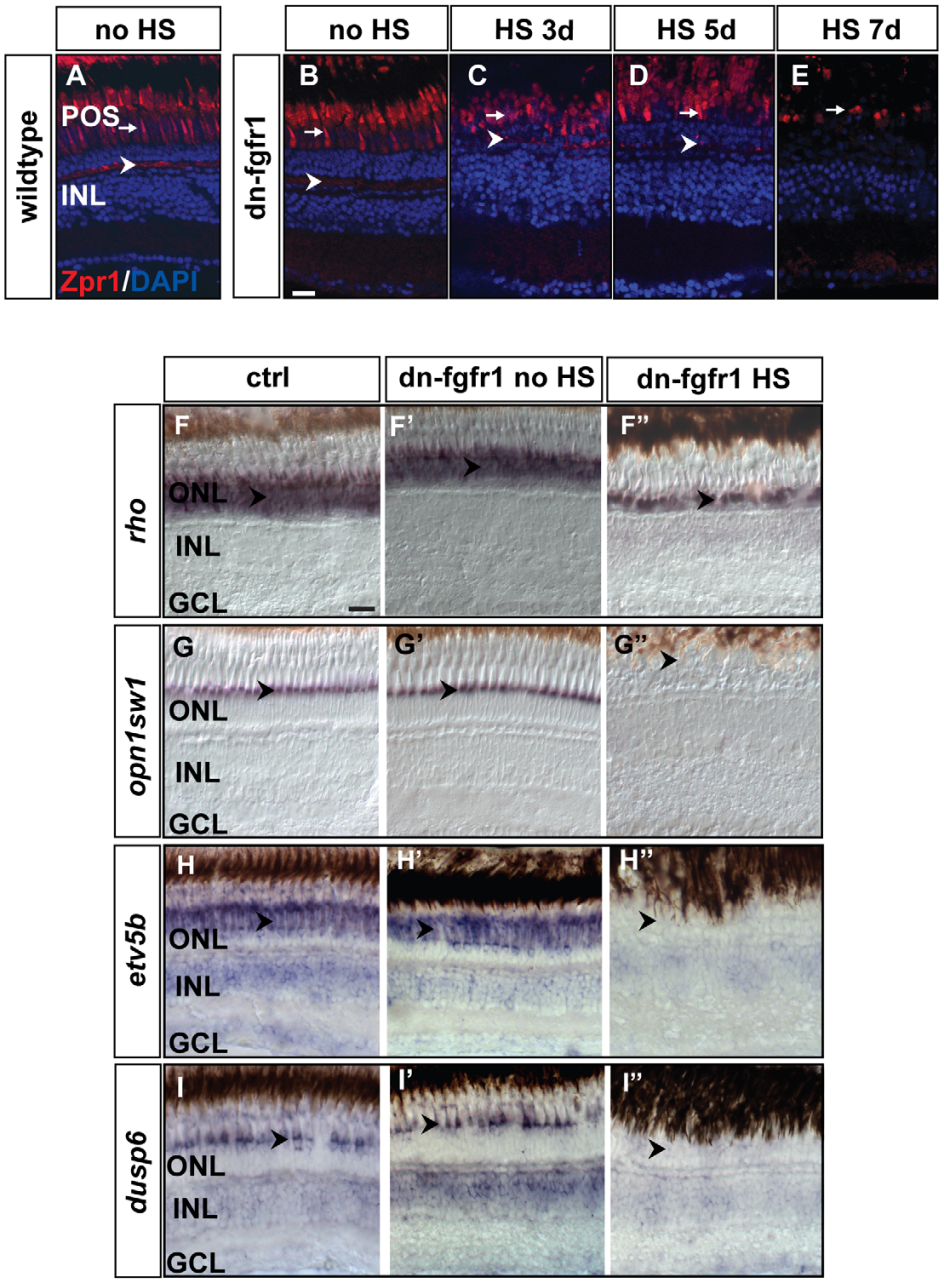
Loss of photoreceptor marker gene expression after Fgf-receptor inhibition. **A**) Expression of the double cone marker Zpr1 in the outer segments (arrow) and in the photoreceptor synaptic terminals (arrowhead) of control siblings after 7 days of HS. **B**) Expression or Zpr1 in transgenic fish without HS (arrow and arrowhead), **C**) after 3 days of HS (arrow and arrowhead), **D**) after 5 days of HS (arrow and arrowhead), and **E**) after seven days of HS. Hardly any photoreceptor marker expression remains visible in outer segments (arrow) and the photoreceptor synaptic terminals are no longer detectable. **F**) The rod photoreceptor marker *rhodopsin (rho)* is expressed in the ONL (arrowhead) of control siblings. **F′**) *rho* expression in the ONL in transgenic non-heatshocked control fish (arrowhead). **F″**) Reduced *rho* expression (arrowhead) in dn-fgfr1 transgenic fish after 5 days of HS. **G**) WT expression of UV opsin (*opn1sw1*) as a cone photoreceptor marker (arrowhead). **G′)** Similar expression of UV opsin in the POS of untreated transgenics (arrowhead). **G″**) Expression of UV opsin is completely absent in transgenic experimental fish (arrowhead) after 2 days of HS. **H**) WT expression of the Fgf signaling downstream target gene *etv5b*. Prominent expression is seen in the POS (arrowhead). **H′**) Comparable expression in untreated dn-fgfr1 fish (arrowhead). **H″**) Complete lack of *etv5b* expression after 2 days of heat shock of dn-fgfr1 fish (arrowhead). **I**) The downstream target *dusp6* is expressed broadly in the retina and prominently in the POS (arrowhead). **I′**) In untreated transgenic fish, a similar pattern as in control siblings was detected with distinct expression in the POS (arrowhead). **I″**) After 2 days of heat shock induction of the dn-fgfr1 transgene, *dusp6* expression is completely lost in the neural retina, including the POS (arrowhead). Scale bars = 20 µm.

To characterize the degeneration of photoreceptor cells further, we used specific markers that label different photoreceptor cell types. We used Zpr1 immunohistochemistry to label red-green double cones, and *in situ* hybridization analyses for *rhodopsin* (*rho*) to detect rod photoreceptors and *opsin 1 (cone pigments), short-wave-sensitive 1* (*opn1sw1*) to distinguish UV cones [12]. In comparison to non-heatshocked transgenic or wild-type siblings, heatshocked dn-fgfr1 fish show a gradual degeneration of Zpr1-positive (Zpr1+) double cones (Fig. 6A–E). Zpr1+ photoreceptors are lost over time and are completely absent by seven days of heat shock treatment (Fig. 6C–E). Notably, the signal at the synaptic terminals of the photoreceptor cells also gets diminished over time and is completely lacking after seven days of treatment. Similarly, *in situ* hybridization analyses shows a diminished expression of *rho* in rod photoreceptors after five days of heat shock in comparison to the wild-type and non-heatshocked control siblings (Fig. 6F–F″). In UV cones, *opn1sw1* is already lost after two days of heat shock treatment in transgenic siblings in comparison to wild-type siblings and non-heatshocked transgenic siblings (Fig. 6G–G″), indicating that UV cones are more sensitive to withdrawal of Fgf signaling. We also investigated the expression patterns of *etv5b* and *dusp6*, which are established downstream target genes of Fgf signaling [33], [34]. *etv5b* and *dusp6* show a profound expression in the ONL, INL and GCL in wild-type siblings and non-heatshocked transgenic siblings. The expression of both genes is strongly diminished within three days of heat shock (Fig. 6H–I″). In summary, our study shows a rapid loss of markers for photoreceptor cells and general loss of Fgf target gene expression after conditionally blocking Fgf signaling.

### The adult neural retina regenerates after Fgf signaling withdrawal induced photoreceptor cell loss

**Figure 7:**
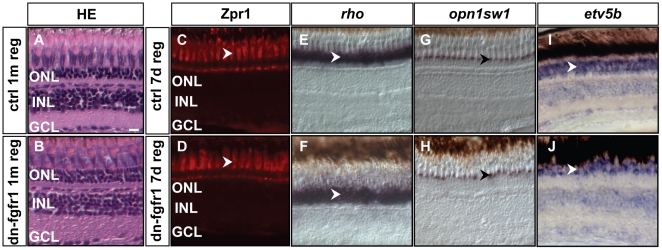
Recovery of retinal tissue architecture and marker gene expression after photoreceptor ablation. **A, B**) Hematoxylin-eosin staining of control retina after one month. dn-fgfr1 transgenic, regenerated retina has recovered a similar layered structure as the retina of wild-type control siblings. **C, D**) Expression of the double cone marker Zpr1 is recovered in the photoreceptor cells of dn-fgfr1 fish (arrowheads) after seven days of regeneration and displays a pattern comparable to control retinas. **E, F**) *rho* expression recovers in transgenic fish and is highly comparable to control fish (arrowheads). **G, H**) UV opsin *(opn1sw1)* expression shows the same pattern in control and in transgenic retinas (arrowheads). **I, J**) Expression of the downstream target gene *etv5b*, indicative of Fgf signaling activity, has recovered in dn-fgfr1 fish after seven days of regeneration and expression is indistinguishable from control fish (arrowheads). Scale bar = 20 µm.

The zebrafish retina is generally able to regenerate following a variety of retinal injuries [19], [35], [36], [37]. We therefore examined whether photoreceptors are able to regenerate following the inhibition of Fgf signaling. We compared the regeneration in transgenic and wild-type siblings by histological analysis. Following photoreceptor ablation from overexpression of the dn-fgfr1 trangene for five days, we examined presence of newly formed photoreceptors one month later. The histological analysis revealed regenerated, layered retinas in the transgenic fish that were comparable to the uninjured wild-type control retinas. Furthermore, the amount and density of cell nuclei in the ONL and the shape of photoreceptor cells were similar between regenerated and control retina (Fig. 7A–B).

To investigate the regeneration process at the level of specific markers, we analyzed photoreceptor-ablated animals after seven days of regeneration. Zpr1 expression in red-green double cones was detected again after seven days of regeneration (Fig. 7C–D). Similarly, *in situ* hybridizations of *rho* and UV opsin (*opn1sw1*) showed fast reappearance of rod photoreceptor and UV cone expression makers in the photoreceptor layer, which resembled the expression pattern in control fish (Fig. 7E–H). Finally, the Fgf downstream target *etv5b* was also prominently re-expressed in its designated domain in the GCL, INL and ONL (Fig. 7I–J). Taken together, our results demonstrated (i) that photoreceptors efficiently regenerated following Fgf signaling withdrawal, (ii) that markers for photoreceptor identity and Fgf downstream targets were re-expressed rapidly due to the regeneration of photoreceptor cells.

**Figure 8:**
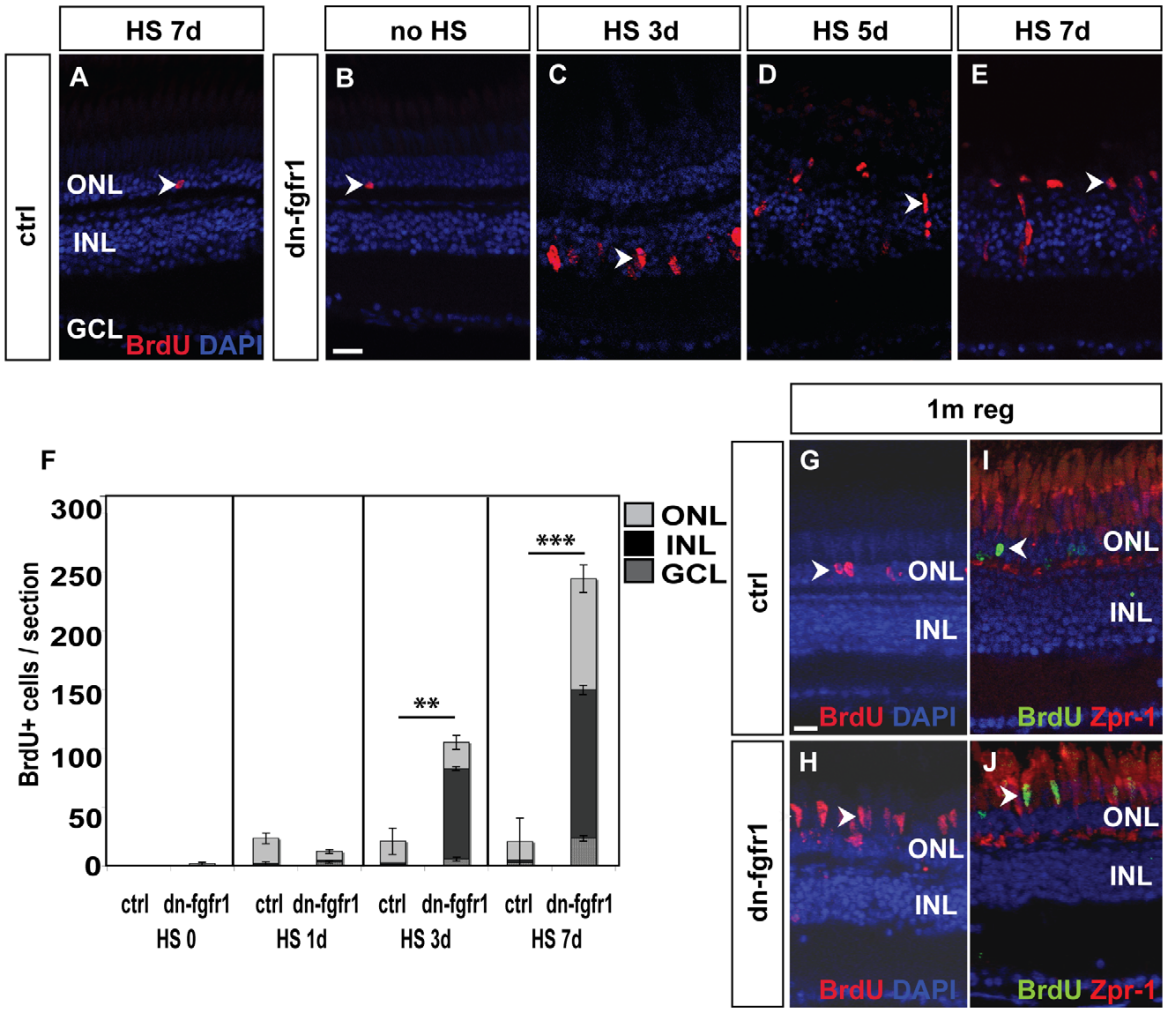
Fgf signaling withdrawal dependent photoreceptor death triggers proliferation response in ONL and INL. **A**) The control retina shows few BrdU+ cells in the ONL (arrowhead). **B**) Non-heatshocked control transgenic fish show similar numbers of BrdU+ nuclei in the ONL (arrowhead) as the control. **C**) After 3 days of heat shock treatment, a strong proliferation response is detectable in the INL (arrowhead). **D**) After five days, cell clusters and fusiform-shaped cells are found in the INL (arrowhead). **E**) After seven days of heat shock induction, a large number of BrdU+ nuclei are located in the ONL (arrowhead). **F**) Quantifications of BrdU+ cells per section. Under control conditions, control siblings and non-treated transgenic fish show hardly any BrdU incorporation. After one day of HS induction, proliferation increases in both groups of experimental fish compared to non-heatshocked controls. There is no significant difference between transgenic and WT siblings (p = 0,11). After three days, many cells proliferate mainly in the INL of transgenic siblings (p = 0,01). At seven days of Fgf signaling inhibition, the number of proliferating cells in the INL and ONL increases even further in dn-fgfr1 fish (p = 4,3E-13). Shown are the mean numbers of BrdU+ nuclei/section. The error bars indicate the SEM. p-values: *≤0.05, **≤0.01, ***≤0.001. **G**) After 5 d of HS, experimental fish were soaked in BrdU, followed by a one month chase time. In control siblings, BrdU+ nuclei were found after one month of regeneration in the ONL (arrowhead). **H**) In transgenic fish elongated BrdU+ cell nuclei, which are characteristic for photoreceptor cells, are detected in the photoreceptor layer (arrowhead). **I**) Zpr1 and BrdU (arrowhead) double positive nuclei were not detectable in control fish. **J**) In contrast, numerous Zpr1 and BrdU double-labeled cells were found in the photoreceptor layer in retinas of transgenic fish (arrowhead). Scale bars = 20 µm.

**Figure 9:**
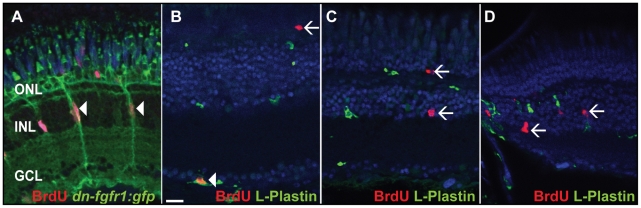
Identification of BrdU+ cells. **A**) Müller glia cells (green) are proliferating in the INL after seven days of HS (white arrowheads). **B**) The vast majority of BrdU+ cells in control and transgenic fish in the GCL colocalize with the pan-leukocyte marker L-Plastin (white arrowhead). **C**) BrdU+ cells in the INL and ONL do not colocalize with L-Plastin in seven days HS control fish (white arrows). **D**) BrdU+ cells in the INL do not colocalize with L-Plastin in transgenic heat shocked fish (white arrows). Scale bar = 20 µm.

To analyze the photoreceptor regeneration response in more detail we used BrdU-labeling experiments to study S phase re-entry of retinal cell types. A time-course experiment allowed us to determine the spatial and temporal dynamics of the proliferation response. In wild-type siblings treated with heat for up to seven days and in non-heatshocked transgenic dn-fgfr1 fish, only very few individual BrdU-positive (BrdU+) cells were located in the ONL or INL (Fig. 8A–B). In contrast, cells started to proliferate and incorporate BrdU in the inner portion of the INL of dn-fgfr1 fish three days after heat shock induction (Fig. 8C); these cells had the typical morphology of Müller glia cells (Fig. 9A). At five days of disrupted Fgf signaling, clusters of fusiform-shaped BrdU+ cells appeared to migrate towards the ONL. Furthermore, a large number of cells were proliferating in the ONL after one week of impaired Fgf signaling (Fig. 8D–E). Quantification of BrdU+ nuclei corroborated a significant number of proliferating cells in the INL, which started at three days and expanded over time. After seven days, a noticeable number of cells were proliferating in the INL as well as in the ONL (Fig. 8F). Moreover we detected a low number of BrdU+ cells in the GCL. To determine the identity of these cells, we used the pan-leukocyte marker L-Plastin. The vast majority of the BrdU+ cells in the GCL colocalize with L-Plastin (Fig. 9B, white arrowhead), suggesting that microglia/macrophages remain in the retina after lesioning. We did not see BrdU+/L-Plastin+ double-positive cells in the INL or ONL of heat shock control (Fig. 9C, white arrows) or transgenic fish (Fig. 9D, white arrows).

To examine whether these newborn cells give rise to photoreceptor cells, we performed long-term BrdU pulse-chase experiments. To this end, we soaked heat shocked dn-fgfr1 transgenic and non-transgenic control fish for two hours in BrdU followed by a chase time of one month. Control retinas displayed BrdU incorporation only in few rods and rod precursor cells of the ONL, as determined by co-labeling with *neuroD* (Fig. S3A–C), whereas numerous BrdU+ nuclei were found in the photoreceptor layer of heat shocked dn-fgfr1 fish. The elongated nuclear shape of these cells suggested they were cone photoreceptors, which we confirmed by co-staining with the double cone marker Zpr1 (Fig. 8G–J). Our results showed that a significant number of cells were stimulated to proliferate in the INL and ONL following temporary Fgf signaling withdrawal and photoreceptor loss. These newly born cells gave rise to numerous newly differentiated cone and rod photoreceptor cells (Figs. 7, 8).

### Fgf signaling is required for proliferation in regeneration

**Figure 10:**
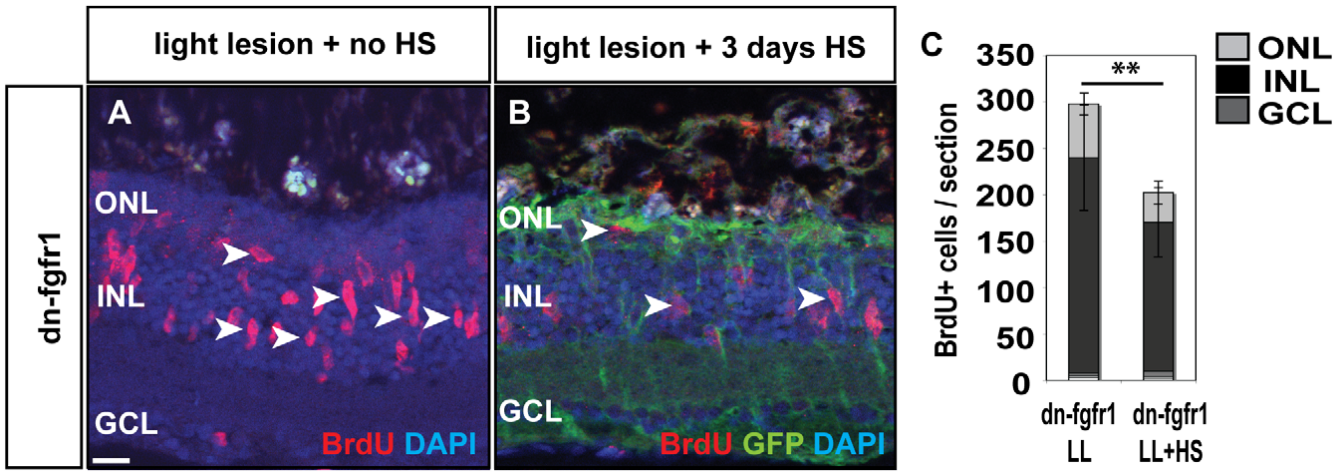
Fgf function in proliferation during regeneration. **A**) Many cells proliferate in the adult retina 3 days after light lesion (white arrowheads). **B**) When Fgf signaling is blocked, proliferation is strongly reduced. **C**) The quantifications show a significant reduction of proliferation after light lesion when Fgf signaling is blocked during the regeneration phase. Scale bar = 20 µm.

To examine a possible influence of Fgf signaling on regeneration, we employed an intense-light-lesion paradigm that specifically depletes photoreceptor cells [36]. In transgenic dn-fgfr1 fish, we introduced a light lesion and subsequently blocked Fgf signaling for three consecutive days via heat shock treatment. As a control, we induced light lesions in the retinas of transgenic siblings that were not heat shocked, and thus had intact Fgf signaling (Fig. 10A). Our results show diminished proliferation in the INL and ONL of the retina when Fgf signaling is blocked (Fig. 10B). We quantified the number of proliferating cells in BrdU-pulse experiments and find a significant decrease of proliferation after injury in fish with impaired Fgf signaling (Fig. 10C). Thus, our results show a significant decrease of proliferating cells in the retina upon impaired Fgf signaling. This indicates that Fgf signaling is required also for proliferation during the regeneration response.

## Discussion

We investigated the role of Fgf signaling in the adult retina of the zebrafish, as an organism that can undergo retinal regeneration. Based on the expression of Fgf ligands, receptors and downstream targets we observe, active Fgf signaling is likely to continously occur in the adult zebrafish retina, notably in photoreceptors and also other layers and cell types. A key finding of our study is that Fgf signaling is required in a relatively selective manner for the maintenance of photoreceptor cells, of both the rod and cone lineage. When Fgf signaling is withdrawn, photoreceptors quickly undergo cell death within a few days. Thus, Fgf signaling is necessary to maintain photoreceptor cells in the adult zebrafish retina, and may be similarly required also in the adult mammalian, including human, retina. In contrast to rodents and humans, zebrafish are capable of regenerating their retina following damage. Following Fgf signaling withdrawal, we observe proliferation in the INL, and BrdU pulse-chase labeling also marks the newly formed photoreceptors, thus confirming the capacity to regenerate photoreceptors also after Fgf signaling withdrawal-induced ablation. Generally, the Fgf signaling withdrawal assay may also be useful as a novel inducible genetic photoreceptor lesion model to study adult retinal regeneration.

### Transgenic dn-fgfr1 line as a tool to study retinal Fgf function

We used a dn-fgfr1 transgenic line (*Tg(hsp70l:dnfgfr1-EGFP*) [20] to study the role of Fgfr-mediated signaling in the adult zebrafish retina *in vivo*. This transgenic line has been used successfully in several zebrafish studies (e.g. heart, fin, brain; [20], [38]). Importantly, this line allows temporally conditional and ubiquitous activation of dn-fgfr1. The Fgf signaling block is thus not confined to the retina or specific retinal cell types. Although we cannot rule out at present that systemic effects might contribute to the observed retinal phenotype, the absence of Fgf target gene expression in the retina upon heat shock induced dn-fgfr1 activation confirms disruption of Fgf signaling in the retina. Further genetic studies using e.g. the Cre^ERT2^/loxP system [39] will be needed to further dissect the tissue and cell type specific requirements for Fgf signaling in the zebrafish retina.

### Fgf signaling in mammalian and zebrafish photoreceptors

Previous studies in rodents and human have detected expression of Fgf pathway components in the retina [40] (and references therein), and suggested a role in cone photoreceptor development [40], [41]. Fgfs are thought to exert neuroprotective effects on mammalian photoreceptors *in vivo* and *in vitro* [42], [43]. The spatial distribution of Fgf pathway components that we observed in the adult zebrafish retina is in agreement with such studies. Blocking Fgf signaling in the mammalian retina has generally led to much milder phenotypes than we observe. In mouse, Campochiaro et al. expressed either dominant negative Fgfr1 or Fgfr2 under the control of the rhodopsin promoter, which led to progressive rod cell death, albeit over a much slower time of months [25], rather than days as observed from zebrafish retinas (this study). Transgenic retinas of one-month-old mice were hardly distinguishable from normal ones. The degeneration of photoreceptors occurred gradually from two months, until around five months of age restricted areas with loss of rods were seen [25]. Our study in zebrafish suggests a more direct dependence of both rod and cone photoreceptors on continued Fgf signaling, because complete photoreceptor loss is observed within one week upon Fgf signaling withdrawal. Apart from unknown species-specific differences, additional criteria likely contribute to this observed difference. For instance, Fgf signaling might be blocked more strongly in the zebrafish transgenic dn-fgfr1 line than in the mouse studies. Indeed, dosage is critical, because we observed that overexpression from a single copy of the dn-fgfr1 transgene produces a much less severe photoreceptor degeneration phenotype than two copies (data not shown). Alternatively, blocking Fgf signaling in the entire retina might have a different effect from blocking it only in photoreceptors, or another cell type. In agreement with this possibility, Rousseau et al. found that overexpressing a dn-fgfr1 construct in developing mouse RPE, a known source of Fgf-2 for photoreceptors [27], caused a loss of up to 50% of all photoreceptors in six-month-old mice. This phenotype is however not getting more severe in 15-month-old animals [27]. In this example, both developmental and RPE-specific Fgf signaling might contribute to ensure photoreceptor survival. Due to the conditional nature of the dn-fgfr1 transgenic line, our study circumvents the well known embryonic developmental requirements for Fgf signaling in the retina (e.g. [44], [45], [46], [47]), and revealed its requirement in adult photoreceptor homeostasis. In teleosts, development of the retina continues life-long in the ciliary marginal zone (CMZ) of the adult retina. We did not observe, however, a difference in BrdU incorporation rates in the CMZ following Fgf inhibition (see Table 1), suggesting that Fgf does not play a major role in the adult CMZ. It remains a possibility that disrupting Fgf signaling in multiple cell types of the central zebrafish retina reveals a more stringent requirement for Fgf in photoreceptors. High resolution Cre ^ERT2^/loxP studies might help to resolve the requirement for Fgf signaling by individual cell types.

**Table 1:** No influence of Fgf inhibition on neurogenesis in the CMZ.

	no. BrdU+ cells (1 d HS)	no. BrdU+ cells (7 d HS)
**ctrl**	11.95 (±1.61)	9.62 (±1.87)
**dn-fgfr1**	10.86 (±5.04)	14.56 (±5.60)

The numbers represent the average of BrdU+ cells in the CMZ (± standard deviation). For this experiment, transgenic and control siblings were heatshocked for one- or seven days, respectively. BrdU-positive cells in the CMZ of both eyes of at least three individuals were counted for each time point.

What is the benefit of continued Fgf signaling to adult photoreceptors? Joly et al. suggested that, in juvenile rats, FGF-2 (and CNTF) might act as a neuroprotective signal for juvenile, but not adult, mammalian photoreceptors. Retinas of juvenile Sprague-Dawley rats have a remarkable intrinsic resistance to light-induced retinopathy, which correlated with overexpression of Fgf-2 [48]. In agreement, we observed a continuous requirement for Fgf signaling, not only during adverse conditions. Nevertheless, continued Fgf signaling in adult retina might similarly increase the likelihood of retaining photoreceptors also under adverse environmental conditions, for instance by continuously reinforcing the differentiated state of photoreceptors.

### Fgf and proliferation during retinal regeneration

In contrast to rodents, the photoreceptor degeneration elicited in the Fgf signaling withdrawal paradigm in zebrafish leads to proliferation and regeneration responses that result in recovery of the lost photoreceptors. During the regeneration phase, cells of the INL start to proliferate three days after onset of Fgf inhibition as soon as the first signs of apoptotic photoreceptor cells become apparent. Previous data from several laboratories have shown that Müller glia cells residing in the INL re-enter the cell cycle and proliferate to give rise to the regenerated cell types [36], [49], and our results suggest that this is also the case after Fgf signaling withdrawal. As regeneration proceeds, we observe BrdU+ nuclei first in the INL and later in the ONL (Fig. 7). In a previous report, ONL proliferation was detected first after light lesions, but spread only secondarily to the INL [35]. This difference in the order of activated progenitor cell types might reflect the differential sensitivity of the photoreceptor subtypes to the lesion paradigm: after Fgf inhibition, cone photoreceptor are more quickly lost than rods (Fig. 6), which may result in more rapid stimulation of MGCs in the INL (Fig. 8), whereas light lesion results in preferential stimulation of rod progenitors in the ONL.

For applications in regenerative biomedicine, it is of great interest to understand the signals that are involved in controlling the regeneration of adult vertebrate photoreceptors. The activity of Fgf pathway genes that we observe in the INL of the non-lesioned retina, and the requirement for Fgf in proliferation following light-lesion, indicates that Fgfs might serve additional roles in the inner retinal layers. For instance, Fgfs might contribute through MGCs in the INL to cell type specification, growth and homeostasis or regeneration, which are exciting possibilities that need further testing.

While this paper was in revision, Qin et al. reported on the role of Fgf signaling in the adult zebrafish retina [50]. Our studies agree on the importance of Fgf signaling in the adult zebrafish retina and suggest a role of Fgf signaling in photoreceptor maintenance. However, differing from our study, Qin et al. report expression only of *fgfr1*, whereas we detected expression of *fgfr1a*, *fgfr2*, *fgfr3* and *fgfr4*, and the expression patterns of Fgf ligands and downstream targets in the adult retina. Furthermore, our results show a fast degeneration of ZPR1+ red-green double cones, *opn1sw1*+ UV cones and *rho*+ rods upon blockage of Fgf signaling (Fig. 6), whereas Qin et al. report degeneration only of rods, not of cones. We tentatively suggest that this difference might reflect the dosage of dn-fgfr1 transgene employed: we studied fish carrying two copies of the dn-fgfr1 transgene (homozygous), whereas Qin et al. studied fish with one copy of the dn-fgfr1 transgene (heterozygous). Indeed, as mentioned above, heterozygous siblings showed weaker photoreceptor degeneration than homozygous animals also in our hands (data not shown). The difference in dn-fgfr1 gene dosage may also explain a difference in the outcome of the regeneration response experiment. While our results show diminished proliferation after light lesion in the INL during Fgf signaling withdrawal (Fig. 10), this is not seen by Qin et al.

## Materials and Methods

### Ethics statement

All experimental procedures were in accordance with the live animal handling and research experimentation regulations of the University and State of Saxony, Germany, review boards (Regierungspräsidium Dresden, permit AZ 24D-9168.11-1/2008-1 and -4). This institutional review board specifically approved this study.

### Fish maintenance

Fish were kept under standard conditions as previously described [51], [52]. Wild-type experimental animals were adult fish from the gol-b1 line in the AB genetic background [53]. Adult fish were 6–8 months old and were 24 mm–32 mm long.

### dn-fgfr1 transgenic line

The dn-fgfr1 transgenic line contains a direct fusion of the dominant negative version of *fgfr1a* to GFP and is under the control of the conditional heat shock 70-like promotor (line kindly provided by Ken Poss); [20]. Heat shock treatment was done at 37°C for 1 h per day. Following heat shock induction of the transgene, both mRNA and GFP protein from the fusion transgene are ubiquitously expressed in the CNS and retina [38].

### BrdU labeling

To label cells in S-phase of the cell cycle, zebrafish were immersed in 10 mM BrdU (Sigma) solution [54]. BrdU was dissolved in E3 medium and adjusted to pH 7.5.

### Tissue preparation and sectioning

Fish heads were fixed at 4°C overnight in 4% paraformaldehyde/0.1 M phosphate buffer (PB), pH 7.5. They were washed twice with 0.1 M PB and transferred for decalcification to 20% sucrose/20% EDTA in 0.1 M PB, pH 7.5. For cryosections heads were frozen in 7.5% gelatine/20% sucrose and sectioned into 14 µm cryosections. Sections were stored at −20°C until use. For paraffin sections processing was done in a Paraffin-Infiltration-Processor (STP 420, Zeiss) according to the following program: ddH_2_0 1×1′; 50% EtOH 1×5′; 70% EtOH 1×10′; 96% EtOH 1×25′; 96% EtOH 2×20′; 100% EtOH 2×20′; xylene 2×20′; paraffin 3×40′/60°C; paraffin 1×60′/60°C. The heads were embedded and 1 µm sections were prepared. The slides were dried on a 37°C heating plate and stored at room temperature until use.

### Immunohistochemistry

Immunohistochemistry was performed as previously described [8], [54]. Primary antibodies were mouse anti-BrdU (1∶500, Becton Dickinson); rabbit anti-GFP (1∶500, Molecular Probes), mouse anti-Zpr1 (1∶500, Developmental Studies Hybridoma Bank, Ames, IA); rabbit anti-activated Caspase-3 (1∶3000, Abcam), mouse anti-HuC/D (1∶300, Invitrogen), rabbit anti-Fgfr1a (1∶200, Anaspec), rabbit anti-Fgfr3 (1∶500, Anaspec), mouse anti-glutamine synthetase (1∶1000, MAB302, Millipore), rabbit anti-L-Plastin (Lcp1, 1∶7500, a kind gift from Michael Redd, University of Utah, Salt Lake City, UT, USA) were used. Briefly, primary and secondary antibodies were incubated in PBS with 0.3% TritonX100 (PBSTx). Primary antibodies were incubated overnight at 4°C and secondary antibodies for 2 h at room temperature. The slides were washed in PBSTx and mounted. The secondary antibodies were Alexa 488-, 555- and 635-conjugated (Invitrogen, Karlsruhe). All immunostainings were done on at least three individuals.

### Specificity of FgfR antibodies

As a control for specificity of FgfR antibodies, blocking peptides (also obtained from Anaspec) for FgfR1a and FgfR3 were included during antibody incubation, which abolished the IHC signal (Fig. S1). The diluted antibody in PBSTx was incubated with 0,1 µg/ml of the respective blocking peptide on a shaker over night at 4°C. As controls, the antibody without blocking peptide and the antibody with a non-corresponding blocking peptide were incubated. After incubation immunohistochemistry was performed as described above. In addition, the FgfR antibodies recognized an IHC signal on adult brain sections that closely resembles the expression pattern detected by ISH (Fig. S2). In sum, we consider it likely that the antibodies indeed recognize FgfR1 and 3, as claimed by the manufacturer.

### *In situ* hybridization

*In situ* hybridization and probe generation was essentially performed as previously described [55]. *In situ* probes for f*gfr1a* [56], *fgfr2*, *fgfr3*, *fgfr4* [57], *fgf8a* [55], *fgf20a* [24], *fgf24* [58], *spry1*, *spry2* [59], *spry4* [60], *etv5a*, *etv5b* [34], *dusp6* [61], *rho* and *neuroD* [12] were used in 1∶100 dilution. All *in situ* hybridizations were done on at least three individuals.

### Light lesion paradigm

Light lesions were performed as previously reported [36].

## Supporting Information

Figure S1 **Specificity test for Fgfr antibodies.** **A**) Fgfr1a antibody staining the GCL, INL and photoreceptor layer (white arrows). **B**) The specific blocking peptide suppresses binding of the Fgfr1a antibody (white arrow). **C**) Fgfr3 antibody staining in the outer part of the INL adjacent to the photoreceptor synaptic terminals (white arrow). **D**) The specific blocking peptide inhibits binding of the Fgfr3 antibody (white arrows). Scale bar = 20 µm.
(TIF)

Figure S2 **Comparison of Fgfr antibody stainings with** ***in situ*** **hybridizations on telencephalic brain sections of adult zebrafish.** **A**) Fgfr1a staining the ventricle (white arrows) **B**) In situ hybridization for *fgfr1a* detectable at the ventricle (black arrows). **C**) Fgfr3 is expressed in the ventricular zone of the dorsal glia domain (white arrow). **D**) Similar expression is detected for *fgfr3* (black arrow).
(TIF)

Figure S3 **Identification of rod progenitors.** **A**) In situ hybridization of *neuroD* shows expression in the ONL in one month chase control fish (white arrows). **B**) BrdU labeling of one month pulse chase fish shows labeling of BrdU in the ONL (white arrows). **C**) The merged picture shows double labeling of some *neuroD+* cells with BrdU (white arrows). *neuroD* labeled cells which do not colocalize with BrdU are also found in the ONL (white arrowhead). Scale bar = 20 µm.
(TIF)
