# Supplementary figures and images for: Fgf Signaling is Required for Photoreceptor Maintenance in the Adult Zebrafish Retina
Source: PLoS One. 2012 Jan 26;7(1):e30365. doi: 10.1371/journal.pone.0030365 (PMC3266925; doi:10.1371/journal.pone.0030365)

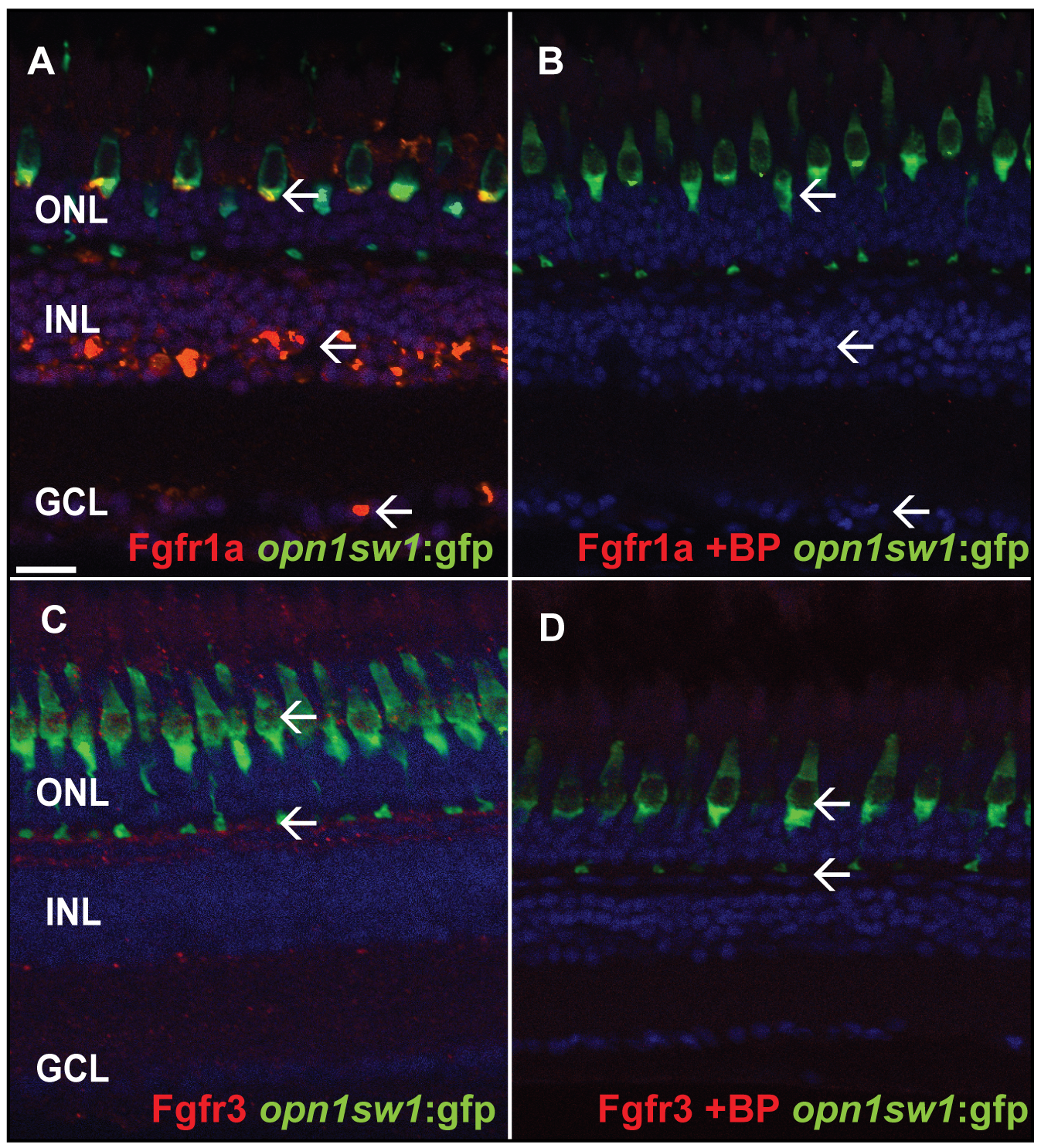

Supplement: Figure S1 — Specificity test for Fgfr antibodies. A) Fgfr1a antibody staining the GCL, INL and photoreceptor layer (white arrows). B) The specific blocking peptide suppresses binding of the Fgfr1a antibody (white arrow). C) Fgfr3 antibody staining in the outer part of the INL adjacent to the photoreceptor synaptic terminals (white arrow). D) The specific blocking peptide inhibits binding of the Fgfr3 antibody (white arrows). Scale bar = 20 µm. (TIF) [file pone.0030365.s001.tif]

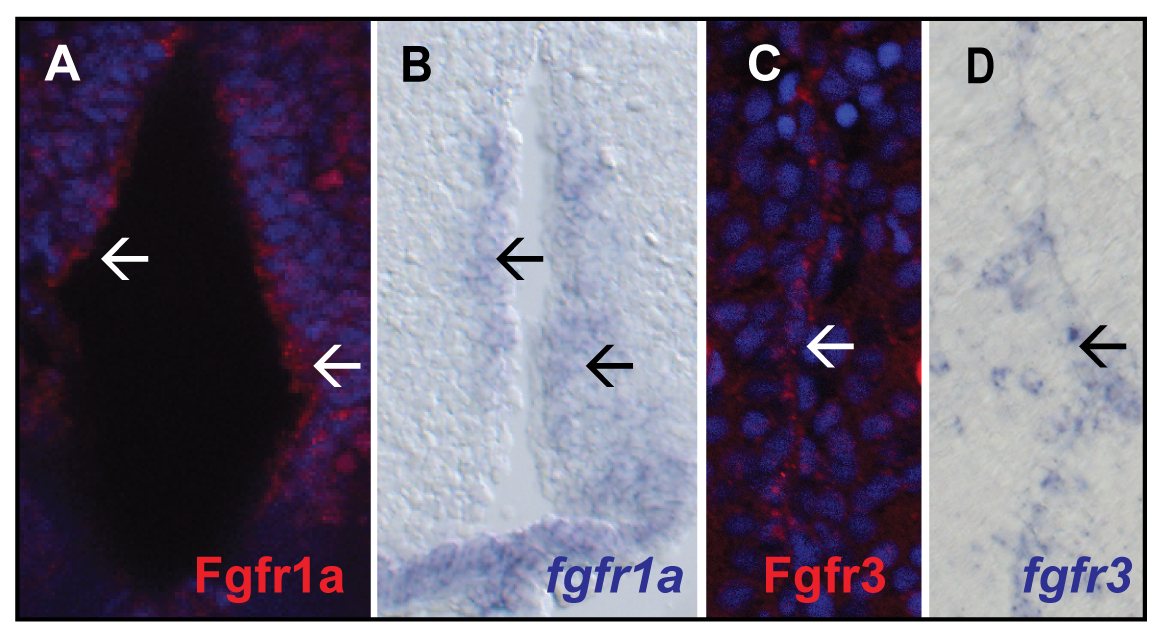

Supplement: Figure S2 — Comparison of Fgfr antibody stainings with in situ hybridizations on telencephalic brain sections of adult zebrafish. A) Fgfr1a staining the ventricle (white arrows) B) In situ hybridization for fgfr1a detectable at the ventricle (black arrows). C) Fgfr3 is expressed in the ventricular zone of the dorsal glia domain (white arrow). D) Similar expression is detected for fgfr3 (black arrow). (TIF) [file pone.0030365.s002.tif]

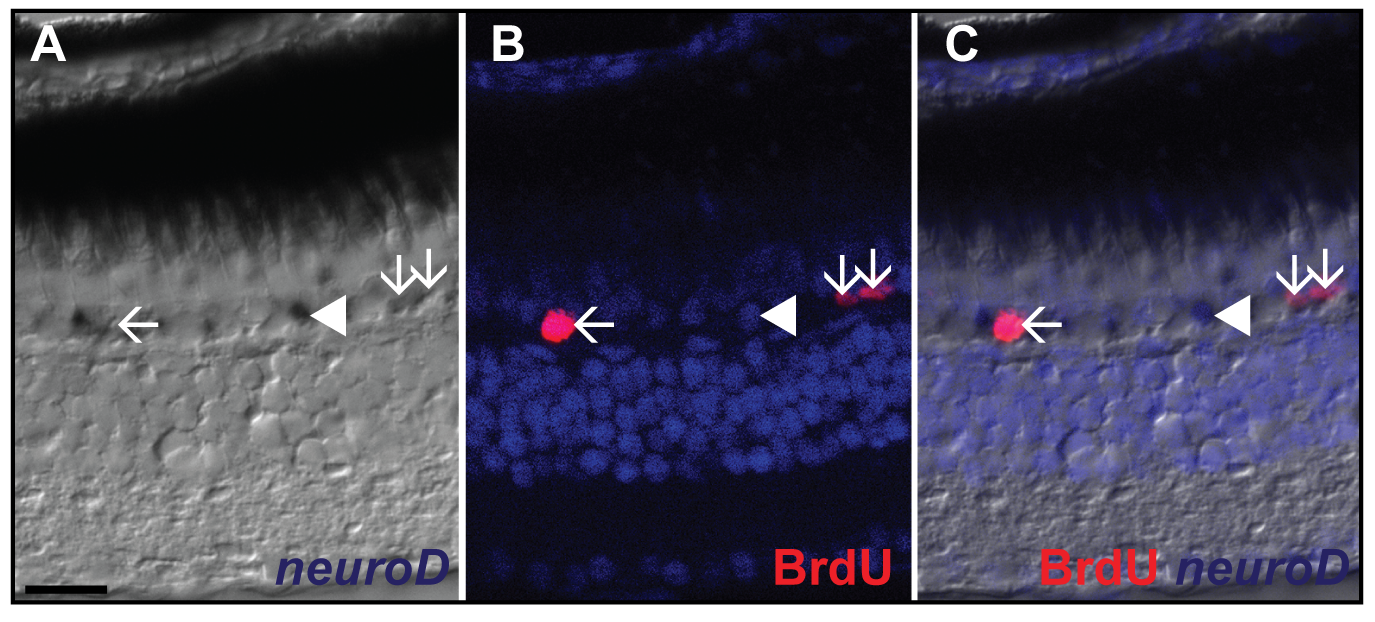

Supplement: Figure S3 — Identification of rod progenitors. A) In situ hybridization of neuroD shows expression in the ONL in one month chase control fish (white arrows). B) BrdU labeling of one month pulse chase fish shows labeling of BrdU in the ONL (white arrows). C) The merged picture shows double labeling of some neuroD+ cells with BrdU (white arrows). neuroD labeled cells which do not colocalize with BrdU are also found in the ONL (white arrowhead). Scale bar = 20 µm. (TIF) [file pone.0030365.s003.tif]
